# Association between brachial-ankle pulse wave velocity and microvascular complications in type 2 diabetes mellitus

**DOI:** 10.1186/s12902-023-01355-w

**Published:** 2023-05-04

**Authors:** Yifei Pei, Yuan Shu, Bo Deng, Yuting Liu

**Affiliations:** 1 Department of Endocrinology, Third Affiliated Hospital of Nanchang University, Nanchang, 330008 Jiangxi Province People’s Republic of China; 2 The Second Clinical Medical College of Nanchang University, Nanchang, 330006 Jiangxi Province China

**Keywords:** Type 2 diabetes mellitus, Brachial ankle pulse wave velocity, Microvascular complications

## Abstract

**Purpose/Aim:**

To investigate the relationship between brachial-ankle pulse wave velocity (baPWV) and microvascular complications in type 2 diabetes mellitus (T2DM).

**Materials and methods:**

From 2019 to 2021, our hospital enrolled 322 patients with T2DM. Clinical information and biochemical indicators of patients were collected from the inpatient electronic medical record system and analyzed retrospectively. Fundus photography, nerve conduction testing, and sensory threshold measurement were all conducted on the subjects. We measured the pulse wave velocity on both sides of the arm and ankle, then averaged the results. The enrolled cases were divided into two groups based on the baPWV: Group 1 (<the reference cutoff value, n = 160) and Group 2 (≥ the reference cutoff value, n = 162). The association between associated factors and baPWV abnormalities was investigated using a logistic regression model.

**Results:**

Group 2 had higher Systolic Blood Pressure(SBP), Diastolic Blood Pressure (DBP), duration of diabetes, Fasting Plasma Glucose (FPG), Blood Urea Nitrogen (BUN) and Serum Uric Acid (SUA) than group 1 (all p < 0.05). The prevalence of diabetic retinopathy, peripheral neuropathy and nephropathy in group 2 was higher compared to group 1 (p < 0.05). After classifying subjects according to the presence or absence of diabetic complications, we found that the baPWV of the Diabetic Peripheral Neuropathy (DPN) group and Diabetic Nephropathy (DN) group was noticeably higher than that of non-DPN group and non-DN group, respectively (both p < 0.05). The baPWV increased with the aggravation of Diabetic Retinopathy (DR) (p < 0.05). Multivariate logistic regression analysis showed that DBP (OR 1.039, 95%CI 1.010–1.068 p = 0.008), duration of diabetes (OR 1.059, 95%CI 1.017–1.103 p = 0.006), FPG (OR 1.104, 95%CI 1.025–1.188 p = 0.009) and BUN (OR 1.128, 95%CI 1.001–1.270 p = 0.048) were all independently and positively associated with baPWV.

**Conclusions:**

The baPWV is strongly associated with microvascular complications of T2DM. The DBP, duration of diabetes, FPG, and BUN were all independent associated factors of baPWV.

## Introduction

Diabetes has become a major global public health concern, with Type 2 Diabetes Mellitus (T2DM) accounting for about 90% of all diabetes [1]. The T2DM is dangerous because its complications endanger the health and life of patients, with Cardiovascular Disease (CVD), Diabetic Nephropathy (DN), diabetic eye disease, and other vascular complications being the leading causes of death and disability of patients [2]. Pulse Wave Velocity (PWV) is a simple, non-invasive and repeatable method for measuring arterial stiffness [3], with brachial-ankle PWV (baPWV) being the most commonly used [4].

The baPWV was found to be a convenient and reliable early indicator for evaluating arterial stiffness, with important clinical significance for predicting cardiovascular and cerebrovascular risk and prognosis in diabetic patients [5]. The higher the PWV, the more severe the arteriosclerosis and the greater the risk of cardiovascular disease. The PWV was also found to be significantly associated with lacunar cerebral infarction [6] and ischemic stroke [7]. Extensive studies have confirmed the relationship between baPWV and macrovascular in T2DM. However, the correlation between baPWV and microvascular complications is rarely discussed. Hence, the objective of the study was to investigate the association between baPWV and microvascular complications in T2DM (Diabetic Peripheral Neuropathy (DPN), Diabetic Nephropathy (DN), Diabetic Retinopathy (DR)) and its risk factors.

## Methods

### Subjects

Between 2019 and 2021, patients with T2DM were enrolled in our Inpatient Department of Endocrinology and Metabolism at the Third Affiliated Hospital of Nanchang University. The inclusion criteria for the subjects were:(1) Age, from 18 to 75 years; (2) T2DM was diagnosed using the 1999 World Health Organization (WHO) diagnostic criteria; (3) Patients volunteered to participate. Participants’ exclusion criteria were (1) severe liver or kidney dysfunction; (2) acute DM complications; (3 ) bilateral lower extremity edema, severe cardiac dysfunction; (4) malignant tumor or pregnancy; (4) cannot cooperate with the examination. In the end, 322 T2DM patients were enrolled. The reference mean values of baPWV in the Central Asian population were used as cut-off values [8]. Both mean and reference values increased with age and blood pressure category. The enrolled cases were divided into two groups: Group 1 (<the reference cutoff value, n = 160) and Group 2 (≥ the reference cutoff value, n = 162). Subjects whose age and blood pressure were classified according to the criteria in the table were assigned to group 2 if their baPWV was higher than the reference mean value to which they belonged, and to group 1 if they were not.(Table 1).

**Table 1:** Reference value of baPWV(m/s) according to age(years) and blood pressure(mmHg)

Age class(years)	Blood pressure class		
	Normal	GradeI	GradeII/III
baPWV as mean(± 2SD)			
< 40	12.3(9.3–15.3)	14.3(10.7–17.9)	15.4(10.8–20)
40–49	12.7(10.1–15.3)	14.1(6.1–22.1)	15.7(10.5–20.9)
50–59	13.9(10.1–17.7)	16.1(10.9–21.3)	17.1(10.7–23.5)
> 60	16.0(10.6–21.4)	18.4(11.8–25.0)	20.3(11.7–28.9)

**Legend**: ***baPWV***, **brachial-ankle pulse wave velocity**

### The collection of data

We obtained basic clinical information from those subjects, such as their name, ages, duration of diabetes, past medical history, and personal history. Furthermore, general vital signs such as blood pressure, weight, and height were routinely measured in patients. The Body Mass Index (BMI) was calculated as weight (kg)/height(m^2^).

Each subject had 3 ml of peripheral venous blood collected and anticoagulated with EDTA. All blood samples were obtained after 8 h of fasting, then analyzed for Fasting Plasma Glucose (FPG), 2-hour Blood Glucose (2 h-BG), glycosylated Hemoglobin (HbA1c), Fasting C-Peptide (FCP), Total Cholesterol (TC), Triglycerides (TG), High-Density Lipoprotein Cholesterol (HDL-C), Low-Density Lipoprotein Cholesterol (LDL-C), Serum creatinine (Scr), Serum Uric Acid (SUA), Blood Urea Nitrogen (BUN), among others.

### Measurement of baPWV

Subjects rested in a quiet room for at least 15 min in a supine position without pillows before baPWV was measured by a professional using an automated atherosclerosis detector (BP-203RPCE, OMRON, Japan). The subject’s hands were placed at the sides of the body, with both upper arms and ankles cuffed. An electrocardiogram sensor was placed in the precordial region, after which the device automatically recorded and analyzed the data. The mean value of baPWV measured on either side of each patient was used for analysis.

### Examination and diagnosis of microvascular complications of T2DM

We used the appropriate methods to diagnose DR, DPN and DN.

According to the International Clinical Grading Criteria for DR, professionals classified patients’ fundus photographic findings as mild, moderate, severe non-proliferate and proliferate [9].

Diagnosis of DPN is made according to the diagnostic basis and diagnostic stratification [10]. Subject history, sensory testing (ankle reflex, temperature sensation, pinprick sensation, pressure sensation), and nerve conduction measurements with the electromyography/evoked potential detection system instrument(CADWELL LABORATORIES, INC), are all part of the DPN examination.

The urinary microprotein and creatinine levels in subjects’ fasting morning urine were determined using dry chemistry, then the urine microprotein to creatinine ratio was calculated. These biochemical indicators are used to identify and classify DN [10]. Moreover, other causes of chronic kidney disease were excluded.

### Statistical analysis

Subjects were divided into two groups based on their baPWV (One group was above the reference cutoff value and the other was below it). Continuous variables have a normal or nearly normal distribution, which is expressed as mean ± standard deviation. For continuous variables, the difference between the two groups was tested using unpaired Student’s test, while X²test was used for categorical variables. Additionally, participants were divided into two groups each time based on the presence of DR, DN or DPN. The two groups were compared by unpaired Student’s test, while one-way ANOVA was used for multiple group measurement data. After entering statistically significant variables data from the t-test into multivariate linear regression analysis, independent risk factors were screened. The Statistical Package for the Social Sciences version 25 (SPSS 25.0) was used for all statistical analyses at *p* < 0.05 or *p* < 0.01.

## Result

### Basic data

A total of 322 T2DM patients were enrolled, with approximately 60% being male(n = 192) and 40% female(n = 130), the mean age was 56.70 ± 12.45 years and the mean baPWV was 15.80 ± 3.24 m/s. After dividing the two groups based on the reference cut-off values, the mean baPWV was 14.21 ± 2.31 m/s in group 1(n = 160) and 17.45 ± 3.18 m/s in group 2(n = 162). There were no statistically significant difference between the two groups (*P* > 0.05) regarding age, sex, smoking, BMI, and alcohol use.There were statistically significant differences between the two groups(*P*<0.05) in anthropometric indices such as FPG, BUN, SUA, systolic blood pressure and diastolic blood pressure (SBP, DBP) (Table 2).

**Fig. 1:**
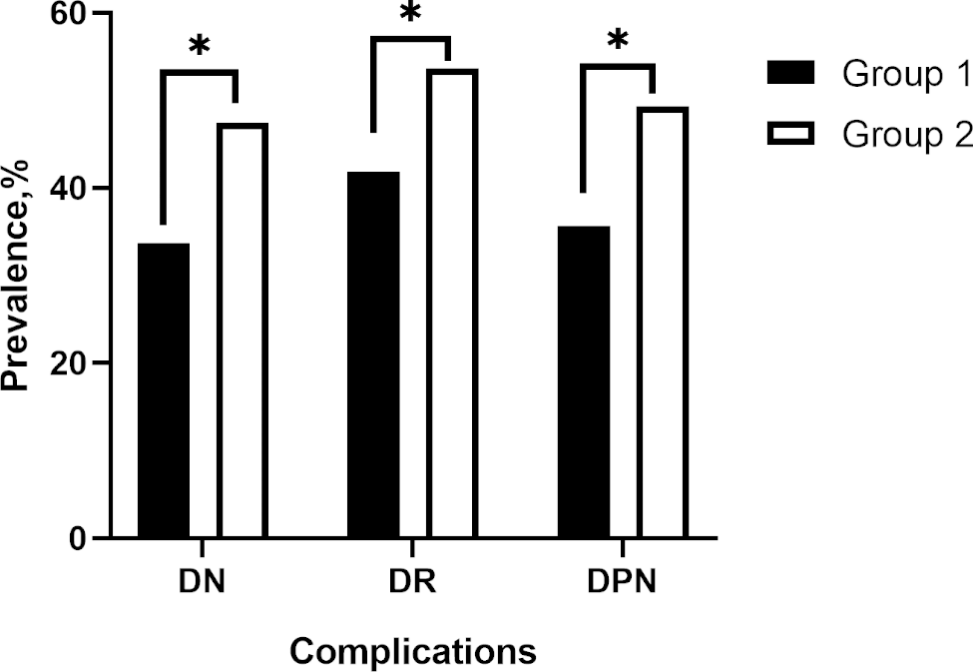
Comparison of the prevalence of the three complications. Legend: Group1(below the reference cutoff value), Group2(above the reference cutoff value). * *P* < 0.05

The prevalence of DR, DPN and DN in group 2 were 53.7%, 49.4%, and 47.5%, respectively, which were higher than 41.8%, 35.6%, and 33.8% in group 1. The differences were statistically significant (Table 2; Fig. 1).

**Table 2:** Basic data and biochemical parameters

	Group1 (n = 160)	Group2 (n = 162)	Total	t/χ22χ2	*P*
baPWV, m/s	14.12 ± 2.31	17.45 ± 3.18	15.80 ± 3.24	−10.70	0.000*
Age,years	56.87 ± 12.52	56.54 ± 12.41	56.70 ± 12.45	0.24	0.811
Gender(M/F)	94/64	96/66	192/130	0.18	0.892
SBP, mmHg	126.40 ± 19.11	135.15 ± 18.91	130.80 ± 19.48	-4.13	0.000*
DBP, mmHg	74.43 ± 10.72	79.93 ± 11.56	77.20 ± 11.47	-4.42	0.000*
BMI, kg/m^2^	25.43 ± 3.39	25.67 ± 4.26	25.55 ± 3.85	-0.56	0.573
Duration of diabetes, years	5.29 ± 5.44	7.28 ± 6.68	6.30 ± 6.17	−2.93	0.004*
Smoking, n(%)	58(36.25)	56(34.57)	114(35.4)	0.10	0.752
Drinking, n(%)	26(16.25)	18(11.11)	44(13.67)	1.80	0.179
Hypertension, n(%)	66(41.25)	40(24.69)	106(32.92)	9.99	0.002*
DR, n(%)	67(41.88)	87(53.70)	154(47.83)	4.51	0.034*
DN, n(%)	54(33.75)	77(47.53)	131(40.68)	6.34	0.012*
DPN, n(%)	57(35.63)	80(49.38)	137(42.55)	6.23	0.013*
Laboratory					
FPG, mmol/L	9.08 ± 3.24	10.13 ± 3.41	9.61 ± 3.36	−2.82	0.005*
2 h-BG, mmol/L	17.86 ± 5.91	18.78 ± 4.99	18.32 ± 5.48	−1.51	0.133
FCP, ng/ml	1.71 ± 1.03	1.79 ± 1.16	1.75 ± 1.09	−0.63	0.532
2 h-CP, ng/ml	4.40 ± 3.08	4.16 ± 2.81	4.28 ± 2.95	0.72	0.470
HbA1c(%)	9.09 ± 2.64	9.32 ± 2.31	9.21 ± 2.48	−8.32	0.406
BUN, mmol/L	5.11 ± 1.86	5.82 ± 3.38	5.47 ± 2.75	−2.34	0.020*
Scr, umol/L	67.32 ± 26.25	71.87 ± 44.49	69.61 ± 36.60	−1.12	0.265
SUA, umol/L	317.13 ± 88.47	339.61 ± 102.78	328.44 ± 96.44	-2.10	0.036*
TG,mmol/L	2.27 ± 2.46	2.63 ± 3.40	2.45 ± 2.97	-1.09	0.276
TC, mmol/L	4.78 ± 1.22	5.09 ± 2.80	4.93 ± 2.16	-1.28	0.202
HDL-C, mmol/L	1.16 ± 0.31	1.17 ± 0.31	1.17 ± 0.31	-0.09	0.927
LDL-C, mmol/L	3.09 ± 0.92	3.18 ± 0.90	3.14 ± 0.91	-1.05	0.294

**Legend: Values are given as the mean ± standard deviation or number (%**), **the test statistic for measurement data is t, the test statistic for the count data is χ2 .*baPWV*brachial-ankle pulse wave velocity**, ***SBP*Systolic Blood Pressure**, ***DBP*Diastolic Blood Pressure**, ***BMI*body mass index**, ***DR*diabetic retinopathy**, ***DPN*Diabetic peripheral neuropathy**, ***DN*diabetic nephropathy**, ***FPG*Fasting Plasma Glucose**, ***2 h-BG*2-hour Blood Glucose**, ***FCP*Fasting C-Peptide**, ***2 h-CP*2-hour C-peptide**, ***HbA1c*glycosylated hemoglobin**, ***BUN*Blood Urea Nitrogen**, ***Scr*Serum creatinine**, ***SUA*Serum Uric Acid**, ***TG*Triglycerides**, ***TC*Total Cholesterol**, ***HDL-C*high-density lipoprotein cholesterol**, *LDL-C*low-density lipoprotein cholesterol. *P **< 0.05, the differences was statistically significant**

### Relationship between baPWV and type 2 diabetic microangiopathy

Each time, the subjects were grouped based on the presence of DR, DN, or DPN, then the differences in baPWV between the groups were compared. The baPWV was significantly higher in the DPN than in the no DPN group, with the difference being statistically significant (*P* = 0.003<0.05) (Table 3a). When grouped by the presence or absence of DN, there was a significant increase in baPWV in the group with DN compared to no DN (*P* = 0.000 < 0.001) (Table 3a). Grouping of subjects according to the stage of DR showed that baPWV in the severe non-proliferative phase group was significantly higher than that in the moderate non-proliferative phase group, baPWV in the moderate non-proliferative phase group was significantly higher than that in the mild non-proliferative phase group, and baPWV in the mild non-proliferative phase group was significantly higher than that in the no DR group (*P* = 0.000 < 0.001). The DR severity increased with the baPWV (Table 3b).

**Table 3a:** Comparison of baPWV(m/s) between subjects with and without DPN and with and without DN

Groups	baPWV, m/s	t	*P*
DPN (n = 137)	16.41 ± 3.18	−2.96	0.003
No DPN (n = 185)	15.34 ± 3.22		
DN (n = 131)	16.80 ± 3.56	−4.57	0.000
No DN (n = 191)	15.11 ± 2.81		

**Legend**: ***P*<0.05, the difference was statistically significant.*baPWV*brachial-ankle pulse wave velocity**, ***DPN*Diabetic peripheral neuropathy**, ***DN*diabetic nephropathy**

**Table 3b:** Grouped according to the stages of DR and compared the baPWV(m/s) with each other

	Normal NPDR (n = 168)	Mild NPDR (n = 101)	Moderate NPDR (n = 34)	Severe NPDR (n = 15)	PDR (n = 4)	F	*P*
baPWV, m/s	15.11 ± 3.04	15.96 ± 3.00	17.38 ± 3.24	18.38 ± 4.23	17.28 ± 4.52	7.07	0.000

**Legend**: ***P*<0.01, the difference was statistically significant.*NPDR*nonproliferative diabetic retinopathy**, ***PDR*proliferative diabetic retinopathy**

**Table 4:** Multivariate logistic regression analysis was performed with baPWV(m/s) and each relevant value

Independent variables	B	OR	(95%CI)	*P*
DBP, mmHg	0.038	1.039	(1.010–1.068)	0.008*
SBP, mmHg	0.011	1.011	(0.995–1.027)	0.193
Duration of diabetes,years	0.058	1.059	(1.017–1.103)	0.006*
FPG, mmol/L	0.099	1.104	(1.025–1.188)	0.009*
BUN, mmol/L	0.120	1.128	(1.001–1.270)	0.048*
SUA, umol/L	0.001	1.001	(0.999–1.004)	0.333

Legend: *P<0.05, the difference was statistically significant.*SBP*Systolic Blood Pressure, ***DBP*Diastolic Blood Pressure**, ***FPG*Fasting Plasma Glucose**, ***BUN*Blood Urea Nitrogen**, ***SUA*Serum Uric Acid**

### Multivariate logistic regression analysis of baPWV and related indexes

Multivariate logistic regression analysis was performed with SBP, DBP, duration of diabetes, FPG, BUN and SUA as independent variables, baPWV (group 1 and group 2) as dependent variables, and group 1 as the reference group. The results showed that DBP{OR = 1.039(1.010–1.068) *P* = 0.008}, duration of diabetes {OR = 1.059 (1.017–1.103) *P* = 0.006}, FPG{OR = 1.104 (1.025–1.188) *P* = 0.009}, and BUN{OR = 1.128(1.001–1.270) *P* = 0.048}were all independently associated with baPWV (Table 4).

## Discussion

In this study we found that a measure of diabetic macrovascular disease - baPWV - was associated with measures of diabetic microvascular disease - eye, kidney, nerve - on cross sectional analysis. These results suggest that the same factors that are associated with macrovascular disease are associated with microvascular disease.

Traditionally, the complications of chronic diabetes mellitus have been primarily considered attributed to vascular injury. However, many researchers have investigated the relationship between arterial stiffness and the occurrence and development of T2DM. BaPWV is a measure of arterial elasticity [11], so we reviewed a large number of articles to analyze the relationship between bapwv and T2DM. In recent years, international studies have found that baPWV is inextricably linked to the development of T2DM as well as the chronic complications that result from it. After adjusting for related influencing factors, baPWV, smoking, and a high LDL-C level were found to be associated with microalbuminuria in T2DM patients [12], implying a possible link between the pathogenesis of atherosclerosis and DN. Arteriosclerosis is a major contributor to the progression of chronic kidney disease [13]. Therefore, some researchers have proposed baPWV as a potential early detection method for DN [14]. Moreover, Siasos et al. found that aortic stiffness determined by carotid-femoral PWV(cfPWV) was significantly associated with DPN [15] in a cross-sectional study. Furthermore, cfPWV is an index of atherosclerosis that is associated with cardiovascular disease risk factors and clinical events to a similar degree as baPWV [16]. After adjustment for several traditional risk factors for DPN development including HbA1c, duration of diabetes, arterial hypertension, dyslipidemia, height, and other microvascular complications like retinopathy and nephropathy, this association remained significant. Concerning DR, An et al. investigated the relationship between atherosclerosis and the progression of DR and concluded that baseline baPWV may be an independent predictor of new onset/progression of DR [17]. According to Liu et al., high PWV (> 19.6 m/s) was strongly associated with the risk of severe DR, particularly PDR [18]. The severity of DR was positively correlated with increasing degree of PWV, generally consistent with the findings of this study.

Moreover, Yiming et al. measured baPWV in a Chinese Xinjiang population of varying age and blood type, the first study of baPWV in a Chinese population [8]. Therefore, the baPWV grouping in this study refers to the reference mean value as the Cut-off value. Subjects were grouped based on whether they had microvascular complications from T2DM. The results also revealed that the baPWV was significantly higher in the affected group, implying that baPWV was associated with microangiopathy in T2DM. These findings are consistent with those of several other researchers.

We investigated the factors that influence baPWV, and discovered that SBP, DBP, duration of diabetes, duration of hypertension, FPG, BUN, and SUA were all positively correlated with baPWV, consistent with the findings of many other researchers. Age and blood pressure are important factors influencing baPWV [19]. Sougawa et al. concluded that the main factors associated with baPWV in both men and women were age, BMI, and blood pressure [20]. Kim found that baPWV increased with age, disease duration, SBP as well as the prevalence of diabetic microvascular complications (DR and DN)[21]. On the contrary, during the baPWV grouping, our study adjusted for two factors, age and blood pressure, increasing the comparability between the two groups.

This study has some limitations and deficiencies. First, because our study was a cross-sectional study, we were unable to judge causality longitudinally. We were also not able to determine the sequence of effects of baPWV, blood pressure, and blood glucose. Second, when collecting clinical data, we did not record a complete medication situation of patients, and hence the statistical results may have been influenced by antihypertensive drugs, hypoglycemic drugs and lipid-lowering drugs.

In conclusion, the baPWV measurement is known to be simple and reliable for determining arterial stiffness [22]. Our findings are consistent with previous studies, implying that baPWV may be a simple and effective indicator to assist in the diagnosis of microvascular diseases in early T2DM.

## Data Availability

All data generated or analyzed during this study are included in this published article.

## Abbreviations

T2DM: type 2 diabetes mellitus
baPWV: brachial-ankle pulse wave velocity
SBP: Systolic Blood Pressure
DBP: Diastolic Blood Pressure
FPG: Fasting Plasma Glucose
BUN: Blood Urea Nitrogen
SUA: Serum Uric Acid
DPN: Diabetic Peripheral Neuropathy
DN: Diabetic Nephropathy
DR: Diabetic Retinopathy
CVD: Cardiovascular Disease
PWV: Pulse Wave Velocity
WHO: World Health Organization
BMI: Body Mass Index
2h-BG: 2-hour Blood Glucose
HbA1c: glycosylated Hemoglobin
FCP: Fasting C-Peptide
2h-CP: 2-hour C-peptide
TC: Total Cholesterol
TG: Triglycerides
HDL-C: High-Density Lipoprotein Cholesterol
LDL-C: Low-Density Lipoprotein Cholesterol
Scr: Serum creatinine
NPDR: nonproliferative diabetic retinopathy
PDR: proliferative diabetic retinopathy
